# Waning Vaccine Protection against Influenza among Department of Defense Adult Beneficiaries in the United States, 2016–2017 through 2019–2020 Influenza Seasons

**DOI:** 10.3390/vaccines10060888

**Published:** 2022-06-01

**Authors:** Wenping Hu, Paul A. Sjoberg, Anthony C. Fries, Laurie S. DeMarcus, Anthony S. Robbins

**Affiliations:** 1 The Department of Defense Global Emerging Infections Surveillance Branch, Armed Forces Health Surveillance Division, Wright-Patterson Air Force Base, Dayton, OH 45433, USA; paul.sjoberg.1.ctr@us.af.mil (P.A.S.); laurie.demarcus.ctr@us.af.mil (L.S.D.); anthony.robbins.5@us.af.mil (A.S.R.); 2 JYG Innovations, LLC, Dayton, OH 45414, USA; 3 U.S. Air Force School of Aerospace Medicine, Wright-Patterson Air Force Base, Dayton, OH 45433, USA; anthony.fries.1@us.af.mil

**Keywords:** influenza, influenza vaccine, vaccine effectiveness, waning, adult

## Abstract

The objective of this study was to assess inactivated influenza vaccine effectiveness (VE) by time since vaccination in adults aged ≥ 18 years using a test-negative design. All data were obtained from the US Department of Defense Global Respiratory Pathogen Surveillance Program over four influenza seasons, from 2016–2017 through 2019–2020. Analyses were performed to estimate VE using a generalized linear mixed model with logit link and binomial distribution. The adjusted overall VE against any medically attended, laboratory-confirmed influenza decreased from 50% (95% confidence interval (CI): 41–58%) in adults vaccinated 14 to 74 days prior to the onset of influenza-like illness (ILI), to 39% (95% CI: 31–47%) in adults vaccinated 75 to 134 days prior to the onset of ILI, then to 17% (95% CI: 0–32%) in adults vaccinated 135 to 194 days prior to the onset of ILI. The pattern and magnitude of VE change with increasing time since vaccination differed by influenza (sub)types. Compared to VE against influenza A(H1N1)pdm09 and influenza B, the decrease of VE against influenza A(H3N2) was more pronounced with increasing time since vaccination. In conclusion, based on the analysis of 2536 influenza-positive cases identified from 7058 adults over multiple influenza seasons, the effectiveness of inactivated influenza vaccine wanes within 180 days after 14 days of influenza vaccination.

## 1. Introduction

Influenza vaccination is considered the most effective way to protect against influenza virus infection. Due to constantly changing influenza viruses, the composition of influenza vaccines is annually updated [1]. The Advisory Committee on Immunization Practices recommends annual influenza immunization for all persons without medical contraindications, starting at 6 months of age [2]. Nevertheless, the immunity derived from annual vaccination is not perfect, and it may wane over the course of an influenza season. In recent years, a number of studies have been conducted in North America [3,4,5,6], Europe [7,8,9,10,11,12,13,14], Asia [15], and Australia [16] to examine the impact of time since vaccination on influenza vaccine effectiveness (VE). It appeared that intra-seasonal waning of influenza vaccine protection occurs, but inconsistent findings were reported among different studies [3,4,12,16]. Such observed discrepancies might be due to differences in temperate/(sub)tropics climate, influenza season, circulating influenza strains, study population, study design, and methodology used among different studies. Further investigations are merited with the aim to gain a better understanding of how influenza VE is associated with time since vaccination within an influenza season.

The Department of Defense (DoD) Global Respiratory Pathogen Surveillance (DoDGRS) Program performs routine laboratory-based respiratory pathogen surveillance. The objective of this study was to use the DoDGRS data from multiple influenza seasons to assess whether there is waning of influenza VE over time since vaccination, and if so, determine the magnitude of waning influenza VE within a single influenza season among DoD adult beneficiaries.

## 2. Methods

### 2.1. Study Population

In the DoDGRS program, patients seeking outpatient care were selected at DoD sentinel or participating sites throughout the United States and around the world. However, the patients in the present study were restricted to adult beneficiaries aged ≥18 years in the United States. The patient selection was based on criteria which meet the influenza-like illness (ILI) case definition. ILI is defined as a patient who exhibits a fever (≥38 °C) and a cough or sore throat that presents within 72 h after illness onset, or a patient who has physician determination as an ILI case.

The specimens were collected by nasopharyngeal wash or swab, processed, and subject to testing using a multiplex respiratory pathogen panel via reverse transcription polymerase chain reaction (RT-PCR), and/or viral culture. Hence, influenza viruses and non-influenza respiratory pathogens were identified and confirmed. Once influenza virus tested positive, the influenza virus (sub)type was further characterized. Patients were considered vaccinated if they received an influenza vaccine 14 days or more before the onset of ILI. Otherwise, patients were considered unvaccinated if they were not vaccinated before the onset of ILI. All immunized patients in the present study received only one dose of influenza vaccine in a given influenza season and were restricted to being vaccinated ≤194 days prior to the onset of ILI. Consistent with the previous study [17], we excluded patients with an unknown vaccination status or type or those vaccinated <14 days prior to the onset of ILI, and patients vaccinated by a vaccine type other than standard-dose inactivated influenza vaccine.

### 2.2. Statistical Analysis

A high ratio of influenza-negative to influenza-positive usually occurred earlier or later in the influenza season. In order to minimize any potential bias due to the high ratio of influenza-negative to influenza-positive, the study was restricted to a range of surveillance weeks from November to April of the following year for the VE analysis, during which approximately 10% or greater influenza positivity rates were achieved.

All DoDGRS data over four influenza seasons from 2016–2017 to 2019–2020 were pooled by influenza season. Analysis was performed using the generalized linear mixed model (GLMM) with logit link and binomial distribution, with influenza season treated as a random effect in the model. Cases were defined as ILI patients with laboratory-confirmed influenza-positive, while controls were defined as ILI patients who tested influenza-negative. The odds of influenza vaccination among cases were compared to the odds of influenza vaccination among controls. The VE was then calculated as: (1 − adjusted odds ratio) × 100%. After initial evaluation of all potential confounding factors, the factors that changed the crude odds ratio by ≥5% were identified. The identified factors, including month of specimen collected, geographic region, and age group, were included in the GLMM models to adjust VE. The time since vaccination within an influenza season was partitioned into three categories (i.e., 14 to 74 days, 75 to 134 days, and 135 to 194 days prior to the onset of ILI), then the statistical models were stratified by the category of time since vaccination. In each of the stratified models, we estimated overall VE against any influenza viruses in the entire adult population and VE by influenza virus (sub)types in separate models (i.e., influenza A(H1N1)pdm09, influenza A(H3N2), or influenza B). A point estimate of VE was considered statistically significant when the lower limit of its associated 95% confidence interval (CI) was greater than zero.

## 3. Results

### 3.1. Patient Characteristics

Across all 4 seasons, a total of 7058 adults aged ≥ 18 years, including 4015 (56.89%) influenza vaccinated and 3043 (43.11%) influenza unvaccinated, were identified for the VE analysis (Table 1). Among these adults, there were 3559 (50.43%) adults aged 18–49 years, 2207 (31.27%) aged 50–64 years, and 1292 (18.31%) aged ≥ 65 years. There were 2536 influenza-positive cases, of which influenza A(H1N1)pdm09, influenza A(H3N2), and influenza B accounted for 792 (11.22%), 905 (12.82%), and 588 (8.33%), respectively, with the remaining being 248 (3.51%) for non-subtyped influenza A and 3 (0.04%) for influenza co-infection (Table 1). Details of patient characteristics for each time since vaccination are shown in Table 1.

### 3.2. VE by Time since Vaccination

Adjusted VE results against laboratory-confirmed influenza among adults aged ≥18 years are shown in Table 2. By time since vaccination, the adjusted VE against any influenza for adults vaccinated 14 to 74 days prior to the onset of ILI was 50% (95% CI: 41–58%) overall, including 49% (95% CI: 33–61%) against influenza A(H1N1)pdm09, 44% (95% CI: 27–57%) against influenza A(H3N2), and 56% (CI: 40–67%) against influenza B. For adults vaccinated 75 to 134 days prior to the onset of ILI, the adjusted VE against any influenza was 39% (95% CI: 31–47%) overall, including 45% (95% CI: 32–55%) against influenza A(H1N1)pdm09, 30% (95% CI: 13–43%) against influenza A(H3N2), and 56% (44–66%) against influenza B (Table 2). For adults vaccinated 135 to 194 days prior to the onset of ILI, the adjusted VE against any influenza was 17% (95% CI: 0–32%) overall, including 30% (95% CI: 4–49%) against influenza A(H1N1)pdm09, 5% (95% CI: −29–30%) against influenza A(H3N2), and 46% (95% CI: 22–63%) against influenza B (Table 2).

## 4. Discussion

We previously conducted the VE analysis among DoD adult beneficiaries aged ≥ 18 years in outpatient settings within the same seasons, regardless of time since vaccination [17]. The dataset used in the previous study had 7114 adults, including all adults involved in the present study and 56 adults who received vaccination ≥195 days prior to the onset of ILI. It was shown that the VE against any influenza in adults was 40% (95% CI: 33–46%) overall, including 46% (95% CI: 36–55%) against influenza A(H1N1)pdm09, 32% (95% CI: 19–42%) against influenza A(H3N2), and 54% (95% CI: 44–62%) against influenza B [17]. The exclusion of those 56 adults in the present study had little impact on estimates of overall VE against any influenza and VE by influenza (sub)types, regardless of time since vaccination.

Here, we characterized the association of effectiveness of inactivated influenza vaccine in adults with increasing time since vaccination within a single influenza season. The adjusted VE against any influenza was observed the highest in adults vaccinated 14 to 74 days prior to the onset of ILI, followed by a VE decline of approximately 8.2% (absolute) per 30 days until 135 to 194 days of vaccination prior to the onset of ILI. By influenza (sub)types, the VE against influenza A(H3N2) decreased considerably, with an approximately 9.9% (absolute) per 30 days declining rate from 14 to 74 days through 135 to 194 days of vaccination prior to the onset of ILI. In contrast, compared to adults vaccinated 14 to 74 days prior to the onset of ILI, the VE estimate was lower but comparable against influenza A(H1N1)pdm09, while it remained unchanged against influenza B for adults vaccinated 75 to 134 days prior to the onset of ILI. Moreover, a greater decline of VE was found in adults vaccinated 135 to 194 days prior to the onset of ILI against influenza A(H1N1)pdm09 versus influenza B, relative to VE in adults vaccinated 14 to 74 days prior to the onset of ILI.

Ferdinands et al. [4] combined data among patients aged ≥9 years over multiple influenza seasons from 2011–2012 through 2014–2015 in the United States, and their analysis showed that VE decreased with increasing time since vaccination, with VE declining rates (absolute) being approximately 7% per month for influenza A(H3N2) and influenza B and 6–11% per month for influenza A(H1N1)pdm09. More recently, Ferdinands et al. [6] observed similar VE declining rates (an absolute decline in VE of approximately 8–9% per month post-vaccination) among influenza-associated hospitalized adults across influenza (sub)types. Moreover, in an I-MOVE European multicenter VE waning study that included data from 2010–2011 through 2014–2015 influenza seasons [12], it was observed that VE against influenza A(H3N2) decreased from 50.6% (95% CI: 30.0–65.1%) at 38 days after vaccination to 0% (95% CI: −18.1–15.2%) at 111 days after vaccination, VE against influenza B declined from 70.7% (95% CI: 51.3–82.4%) at 44 days after vaccination to 21.4% (95% CI: −57.4–60.8%) at day 207 after vaccination, while VE against influenza A(H1N1)pdm09 reached 55.3% (95% CI: 37.9–67.9%) at 54 days after vaccination and remained unchanged until the end of the influenza season. In the present study, it is clearly shown that the adjusted influenza VE against any influenza decreased with increasing time since vaccination within 180 days, since 14 days of vaccination. A recent meta-analysis which compared VE 15–90 days versus 91–180 days after vaccination confirmed a significant decline of VE in the 91–180 days following vaccination [18]. By comparison with the findings from those studies [4,6,12,18], it appeared that the waning of VE against influenza A(H3N2) was most pronounced, but the pattern and magnitude of waning VE against influenza A(H1N1)pdm09 and influenza B with increasing time since vaccination varied among the different studies.

The waning effect of the influenza vaccine over time in a given influenza season may be related to changes in the host immune response, circulating influenza viruses, or a combination of both [19]. Protection against influenza virus infection is primarily mediated via antibody responses following influenza vaccination. It was found that hemagglutination-inhibiting antibody titers, as measured by the hemagglutination inhibition assay to indicate the antibody induced by influenza, were associated with clinical protection against influenza virus infection [20]. However, the hemagglutination-inhibiting antibody titers decreased over time since vaccination [21,22,23]. Recently, Davis et al. [24] examined the production and maintenance of bone marrow plasma cells by which long-term serum antibody levels are maintained in adults, and found that the numbers of influenza-specific bone marrow plasma cells increased four weeks after influenza vaccination but returned to near to pre-vaccination levels after one year. Their findings suggest that the most vaccine-induced bone marrow plasma cells cannot be maintained after a certain period of time [24]. Indeed, the immune response to influenza vaccination is short-lived [25,26]. Influenza viruses can escape from the immunity acquired by vaccination over time owing to their high mutation rates and antigenic flexibility [25]. Nevertheless, the possible waning of influenza VE as a result of changes of circulating influenza viruses throughout a single influenza season is not well-understood. Ferdinands et al. [6] observed the intra-seasonal waning of VE among hospitalized adults, but with minimum antigenic drift among influenza viruses circulating during the influenza seasons they analyzed. The change of circulating influenza viruses can occur anytime during an influenza season. However, it may be reasonable to expect that there is antigenic drift accumulation toward the end of influenza season, which would cause waned VE with increasing time since vaccination. Further research is needed to characterize influenza antigenic evolution and distribution in a single influenza season and to clarify to what extent the waning VE is due to influenza antigenic drift over time.

Immune responses to influenza vaccination may differ by age. We pooled data from four influenza seasons to increase the available sample size. Even so, the sample size remained limited, which would not allow to accurately estimate age-stratified VE by time since vaccination, specifically for adults aged ≥65 years. In addition, we were unable to collect data on the past history of vaccine status and influenza virus infection in previous seasons; thus, the models used in the present study did not account for such confounders to adjust VE estimates. Further, the lack of this information prevented an effectiveness analysis of the influenza vaccine over time, by categorizing prior exposures to influenza virus antigens via repeated influenza vaccination or natural influenza virus infection in previous seasons in separate models. It is worth mentioning in relation to methodological issues in a test-negative design for the VE waning study [27]. In the VE waning study, depletion-of-susceptibilities bias may arise when higher-risk patients are depleted from the at-risk population at different rates between the unvaccinated and vaccinated groups, which can make waning appear to occur even if VE does not wane over time [28]. Ray et al. [28] re-analyzed the data from their previous study [29] by focusing on individuals who were vaccinated before influenza was circulating to avoid the depletion-of-susceptibilities bias, and found that the waning effect was evident, and similar to that observed previously.

The finding of intra-seasonal influenza VE waning provides a challenge for public health policy. This highlights a need to determine optimal timing of vaccination, a strategy that can delay the intake of vaccines until a time closer to the predicted beginning of an influenza epidemic in an influenza season, while the immunization rate is minimally compromised. Recently, an effort has been made to evaluate the impact of modifying the timing of influenza vaccination among older adults in the United States, and it was found that the consequences of delayed vaccination varied widely, depending heavily on influenza season timing, the rate of waning, and the overall VE [30]. The impact and magnitude of waning is yet to be established. Caution should be taken to approach any changes in recommendations regarding the timing of influenza vaccination [29].

## 5. Conclusions

Our study adds to growing evidence suggesting that the effectiveness of inactivated influenza vaccines wanes within a single influenza season. The immune response to influenza vaccines over time is complex, with many contributing factors, such as circulating influenza (sub)type in a given season, age group, and past history of vaccination status or natural influenza infections in previous seasons. Future research is warranted to explore how those circulating influenza viruses and host factors influence changes of immune responses and VE against influenza viruses over the course of a single influenza season.

## Figures and Tables

**Table 1:** Characteristics of the study population used for vaccine effectiveness analysis.

	Overall	14–74 Days ^b^	75–134 Days ^b^	135–194 Days ^b^
Characteristic	*n* (%)	*n* (%)	*n* (%)	*n* (%)
Gender				
Male	2341 (33.17)	1291 (31.51)	1637 (32.26)	1243 (31.29)
Female	4717 (66.83)	2806 (68.49)	3437 (67.74)	2730 (68.71)
Age				
18–49 years	3559 (50.43)	2330 (56.87)	2719 (53.59)	2234 (56.23)
50–64 years	2207 (31.27)	1242 (30.31)	1587 (31.28)	1224 (30.81)
≥65 years	1292 (18.31)	525 (12.81)	768 (15.14)	515 (12.96)
Influenza season				
2016–2017	767 (10.87)	532 (12.99)	606 (11.94)	523 (13.16)
2017–2018	1674 (23.72)	1106 (27.00)	1261 (24.85)	1007 (25.35)
2018–2019	2370 (33.58)	1106 (27.00)	1567 (30.88)	1295 (32.60)
2019–2020	2247 (31.84)	1353 (33.02)	1640 (32.32)	1148 (28.90)
Month of illness				
November	231 (3.27)	228 (5.57)	166 (3.27)	163 (4.10)
December	975 (13.81)	850 (20.75)	599 (11.81)	480 (12.08)
January	1925 (27.27)	1257 (30.68)	1521 (29.98)	909 (22.88)
February	2007 (28.44)	962 (23.48)	1615 (31.83)	1066 (26.83)
March	1555 (22.03)	629 (15.35)	980 (19.31)	1032 (25.98)
April	365 (5.17)	171 (4.17)	193 (3.80)	323 (8.13)
Geographic region ^a^				
Region 1	23 (0.33)	12 (0.29)	20 (0.39)	13 (0.33)
Region 2	476 (6.74)	316 (7.71)	376 (7.41)	312 (7.85)
Region 3	484 (6.86)	333 (8.13)	385 (7.59)	336 (8.46)
Region 4	923 (13.08)	651 (15.89)	732 (14.43)	618 (15.55)
Region 5	985 (13.96)	382 (9.32)	589 (11.61)	364 (9.16)
Region 6	2535 (35.92)	1318 (32.17)	1745 (34.39)	1344 (33.83)
Region 7	245 (3.47)	177 (4.32)	196 (3.86)	148 (3.73)
Region 8	677 (9.59)	460 (11.23)	494 (9.74)	427 (10.75)
Region 9	467 (6.62)	299 (7.30)	364 (7.17)	266 (6.70)
Region 10	243 (3.44)	149 (3.64)	173 (3.41)	145 (3.65)
Month of vaccination				
August	25 (0.62)	0 (0.00)	4 (0.10)	21 (0.52)
September	421 (10.49)	16 (0.40)	185 (4.61)	220 (5.48)
October	1818 (45.28)	255 (6.35)	975 (24.28)	588 (14.65)
November	1224 (30.49)	427 (10.64)	698 (17.38)	99 (2.47)
December	353 (8.79)	193 (4.81)	158 (3.94)	2 (0.05)
January	135 (3.36)	124 (3.09)	11 (0.27)	0 (0.00)
February	37 (0.92)	37 (0.92)	0 (0.00)	0 (0.00)
March	2 (0.05)	2 (0.05)	0 (0.00)	0 (0.00)
Vaccine status				
Vaccinated	4015 (56.89)	1054 (25.73)	2031 (40.03)	930 (23.41)
Unvaccinated	3043 (43.11)	3043 (74.27)	3043 (59.97)	3043 (76.59)
Influenza				
A(H1N1)pdm09	792 (11.22)	499 (12.18)	620 (12.22)	485 (12.21)
A(H3N2)	905 (12.82)	582 (14.21)	699 (13.78)	574 (14.45)
A/not subtyped	248 (3.51)	102 (2.49)	175 (3.45)	147 (3.70)
B	588 (8.33)	423 (10.32)	480 (9.46)	405 (10.19)
Dual influenza	3 (0.04)	2 (0.05)	3 (0.06)	2 (0.05)
Non-influenza	4522 (64.07)	2489 (60.75)	3097 (61.04)	2360 (59.40)

^a^ The US Health and Human Services Regions 1–10, except for Guam, Alaska, and Hawaii. ^b^ Time since vaccination prior to the onset of ILI.

**Table 2:** Adjusted vaccine effectiveness in adults aged ≥ 18 years.

	Unvaccinated		Vaccinated			
	Total	Case (%)	Total	Case (%)	VE (%) ^a^	95% CI (%)
14–74 days ^b^						
Influenza A(H1N1)pdm09	2118	406 (19.17)	870	93 (10.69)	49	33–61
Influenza A(H3N2)	2187	475 (21.72)	884	107 (12.10)	44	27–57
Influenza B	2072	360 (17.37)	840	63 (7.50)	56	40–67
Any influenza ^c^	3043	1331 (43.74)	1054	277 (26.28)	50	41–58
75–134 days ^b^						
Influenza A(H1N1)pdm09	2118	406 (19.17)	1599	214 (13.38)	45	32–55
Influenza A(H3N2)	2187	475 (21.72)	1609	224 (13.92)	30	13–43
Influenza B	2072	360 (17.37)	1505	120 (7.97)	56	44–66
Any influenza ^c^	3043	1331 (43.74)	2031	646 (31.81)	39	31–47
135–194 days ^b^						
Influenza A(H1N1)pdm09	2118	406 (19.17)	727	79 (10.87)	30	4–49
Influenza A(H3N2)	2187	475 (21.72)	747	99 (13.25)	5	−29–30
Influenza B	2072	360 (17.37)	693	45 (6.49)	46	22–63
Any influenza ^c^	3043	1331 (43.74)	930	282 (30.32)	17	0–32

^a^ VE: vaccine effectiveness, adjusted for months of specimen collected, geographic region, and age groups. ^b^ Time since vaccination prior to the onset of ILI. ^c^ Including influenza A(H1N1)pdm09, influenza A(H3N2), influenza B, influenza A/not subtyped, and influenza co-infection.

## Data Availability

All data are presented in this article. For further information, contact the corresponding author.
